# Readability of Online Informed Consent Forms for Implant-Based Breast Augmentation Across 6 Languages: Cross-Sectional Study

**DOI:** 10.2196/101399

**Published:** 2026-09-02

**Authors:** Klaudia Kasperska, Tomasz Skrzypczak, Paweł Gać

**Affiliations:** 1 Specialist Medical Center, Polanica Zdrój, Poland, ul. Jana Pawła II, 2, Polanica Zdrój, 57-320, Poland, 48 505332517; 2 ul. Szpitalna 14, Prudnik Medical CenterPrudnik, Poland; 3 Division of Environmental Health, Occupational Medicine and Epidemiology Home page: Division of Environmental Health, Occupational Medicine and Epidemiology Department of Environmental Health, Occupational Medicine and Epidemiology, Wroclaw Medical University, Wroclaw, Lower Silesia, Poland

**Keywords:** informed consent, readability, breast augmentation, internet research, health literacy

## Abstract

**Background:**

Breast augmentation is one of the most performed aesthetic surgical procedures worldwide. Given its elective character and potential complications, clear and comprehensible informed consent is essential.

**Objective:**

The aim of this paper is to evaluate and compare the readability of online informed consent forms for implant-based breast augmentation in languages used in the countries with the highest procedural volumes according to the 2024 International Society of Aesthetic Plastic Surgery (ISAPS) survey.

**Methods:**

The phrase “breast augmentation consent form” was translated into 8 languages and searched using Google in private mode. After applying exclusion criteria, 77 documents were analyzed. Readability was assessed using the *Läsbarhetsindex* (LIX) index.

**Results:**

The overall mean LIX score was 53 (SD 9), corresponding to very difficult texts. Significant differences in readability were observed between languages (*P*<.001). English-language forms demonstrated the lowest mean LIX score (46, SD 7), classified as difficult, whereas Portuguese (55, SD 9), Italian (58, SD 6), and Turkish (63, SD 2) were assessed as very difficult or highly complex. No document was classified as easy or moderately difficult. There were no statistically significant differences in readability between private and nonprivate practice sources, nor was there any correlation between the number of available forms and mean LIX values.

**Conclusions:**

Online consent forms consistently exhibit high linguistic complexity, underscoring the need for systematic simplification and standardization.

## Introduction

Breast augmentation with implants is one of the most performed aesthetic surgical procedures worldwide. According to the 2024 International Survey on Aesthetic/Cosmetic Procedures published by the International Society of Aesthetic Plastic Surgery (ISAPS), breast augmentation accounted for approximately 1,658,615 procedures globally, confirming its sustained popularity across continents [1]. The highest national procedure volumes were reported in Brazil (n=232,593, 14.0%) and the United States (n=227,470, 13.7%), followed by Italy (n=74,392, 4.5%), Germany (n=70,128, 4.2%), Mexico (n=67,893, 4.1%), France (n=58,520, 3.5%), Turkey (n=48,179, 2.9%), India (n=47,600, 2.9%), Spain (n=42,129, 2.5%), and Colombia (n=33,984, 2.0%) [1]. Collectively, these 10 countries account for a substantial proportion of all breast augmentation procedures performed worldwide, underscoring the global relevance of patient communication and informed consent across diverse linguistic and cultural settings.

Breast augmentation is pursued for a variety of patient-centered reasons, including restoration of breast volume following pregnancy or weight loss, correction of congenital or acquired asymmetry, enhancement of body proportion, and improvement of self-image and psychosocial well-being. Previous studies have demonstrated that motivation for breast augmentation was typically multifactorial and primarily internally driven rather than resulting from external pressure, with many patients reporting long-standing dissatisfaction with breast appearance and expectations of improved self-confidence and quality of life [2-4]. Patient-reported outcome research showed significant postoperative improvements in satisfaction with breasts and psychosocial and sexual well-being following breast augmentation [5]. Nevertheless, these benefits should be weighed against potential risks.

Implant-based breast augmentation has been associated with both short- and long-term complications, including infection, hematoma, seroma, implant malposition, rupture, and capsular contracture, the latter remaining one of the most frequent indications for revision surgery [6,7]. Moreover, breast implants are not lifetime devices, and patients should be aware of the potential need for additional operations over time [6]. The presence of such device-related and time-dependent risks highlights the critical importance of comprehensive patient counseling.

In this context, informed consent plays a central ethical and clinical role. Valid informed consent requires that patients clearly understand the expected benefits of surgery, the available alternatives, and the full range of possible complications and long-term consequences [8]. However, multiple studies across medical and surgical specialties have shown that informed consent documents often exceed recommended reading levels, thereby limiting patient comprehension and undermining shared decision-making [9,10]. Similar concerns have been raised within plastic surgery, including analyses of online patient education materials related to breast augmentation [11].

Accordingly, the aim of the present study was to evaluate the readability of publicly available online informed consent documents, which represent one of the information resources that prospective patients could access before a surgical consultation. This study was not intended to evaluate institution-specific consent forms routinely used during the formal clinical informed consent process. Publicly available online consent documents for implant-based breast augmentation in the languages of the 10 countries with the highest procedural volumes worldwide according to the 2024 ISAPS survey (Brazil, United States, Italy, Germany, Mexico, France, Turkey, India, Spain, and Colombia) were included [1]. To our knowledge, no previous study has systematically evaluated and compared the readability of breast augmentation informed consent documents across multiple high-volume countries and languages despite the worldwide popularity of this procedure and the increasing reliance on online health information.

## Methods

### Search Method

Google Translate was used to translate the phrase “breast augmentation consent form” into the languages of the countries where breast augmentation procedures were most frequently performed according to the ISAPS. Google Translate was selected because it provided a standardized and reproducible translation approach across all included languages, ensuring that the same translation methodology was applied consistently throughout this study. No formal validation of the translated search terms by native speakers or professional translators was performed. Each translated phrase was entered into a new Google search session, and searches were performed. To maximize the reliability of the searches, private browsing mode was used, and the search language was changed to the language used in the country in question. All searches were performed from a single geographic location using the same computer, internet connection, browser configuration, and private browsing mode to standardize the search conditions across all languages. No virtual private network or regional location simulation tools were used. All searches were performed between January 1st and February 15th, 2026. The first 50 Google search results were included because the objective of this study was to evaluate the informed consent forms most likely to be encountered by prospective patients during routine internet searches rather than to comprehensively identify all publicly available documents. Previous research has consistently demonstrated that users predominantly interact with the highest-ranked search results, with only a small minority proceeding to later pages of search results [12-14]. Consequently, extending the search to more than 50 results would have addressed a different research question by increasing the number of low-visibility documents rather than improving the representativeness of materials typically accessed by patients [12-14]. Therefore, a sensitivity analysis using more than 50 search results was not performed, as it was not aligned with the predefined objective of this study [12-14].

The highest numbers of breast augmentations are performed in the following countries: Brazil, the United States, Italy, Germany, Mexico, France, Turkey, India, Spain, and Colombia [1]. For this reason, the search covered the following languages: Portuguese, English, Italian, German, Spanish, French, Turkish, and Hindi. Because Spanish is the official language of Spain, Mexico, and Colombia and changing the location in the Google search did not change the search results, the research for these countries was conducted together. Table 1 presents the search term translated in each language. Results in languages other than the search language, pages that were pay-walled, and password-protected pages were excluded from this study. Only consent forms that were publicly accessible through internet search engines were eligible for inclusion. These documents were analyzed as publicly available patient information resources. This study did not attempt to determine whether the retrieved forms were identical to those routinely used during the clinical informed consent process in individual institutions.

**Table 1.** Translations of search term used in this study.

Language	Translation
Portuguese	formulário de consentimento para aumento de mama
English	breast augmentation consent form
Spanish	formulario de consentimiento para aumento de senos
Italian	modulo di consenso per l’aumento del seno
Turkish	meme büyütme ameliyatı onay formu
French	formulaire de consentement pour l’augmentation mammaire
German	Einverständniserklärung zur Brustvergrößerung
Hindi	स्तन वद्ृधि सहमतत पत

### Readability Assessment

The *Läsbarhetsindex* (LIX) index was used to assess the readability of all included materials based on average sentence length and the percentage of long words (more than 6 letters) [15,16]. The LIX score was computed according to the original LIX formula: LIX = (number of words/number of sentences) + (number of long words × 100/number of words), where long words are defined as words containing more than 6 letters [15]. The LIX index is a recognized tool for assessing text difficulty and is used in multilingual studies, including analyses of medical materials and health information available on the internet [17]. Although LIX does not capture all dimensions of readability, it was selected because it enables standardized comparisons across typologically different languages without requiring language-specific syllabification algorithms, making it particularly suitable for multilingual readability studies [15,17]. The analyzed text was copied into Microsoft Word, and nonsubstantive elements (eg, hyperlinks, affiliations, figure captions, and author and copyright information) were removed. The files were saved as plain text. Each text file was then analyzed using an online LIX calculator [18], which automatically computes LIX scores according to the original LIX formula. The number of sentences, the number of words, and the final LIX score were recorded and interpreted according to the original LIX classification proposed by Björnsson [19], which is also implemented in the online LIX calculator used in this study. This study focused exclusively on the linguistic readability of the included consent forms. No qualitative assessment of content completeness, accuracy, or compliance with informed consent recommendations was performed.

### Source Classification

Sources classified in this study as “private practice” refer to breast augmentation consent forms published online by for-profit private plastic surgery clinics that focus on aesthetic plastic surgery. All other sources, such as government websites, scientific societies, university hospitals, and implant manufacturers, were classified as “nonprivate practice.”

### Statistical Analyses

Descriptive statistics are presented as mean (SD). Additionally, a 95% CI was calculated for the mean LIX score of each language group to estimate the precision of the language-specific readability estimates. Differences between languages were assessed using 1-way ANOVA. Given the unequal sample sizes across language groups, Welch ANOVA was additionally performed as a robustness analysis. Homogeneity of variances was assessed using the Levene test, while the normality of variable distributions was evaluated using the Shapiro-Wilk test. A value of *P*≤.05 was considered statistically significant. Statistical analyses were performed using JASP (version 0.98.1; JASP Team, University of Amsterdam), while Microsoft Word and Microsoft Excel (version 16.59; Microsoft Corp) were used for data organization and aggregation.

### Ethical Considerations

This study was based exclusively on publicly available online informed consent documents and did not involve human participants, patient data, or identifiable personal information. Therefore, ethics committee approval and informed consent were not required.

## Results

### Prevalence

Overall, 77 consent forms in 8 languages from 8 countries were found for this analysis. There was only 1 form in German and 1 in Hindi; thus, those languages were excluded from the study. The most numerous consent forms were in Portuguese (n=25, 32.5%), English (n=21, 27.3%), and Spanish (n=17, 22.1%). The majority (n=64, 83.1%) of the included consent forms were published by private practices, followed by other, less numerous sources. The details are presented in Table 2.

**Table 2.** Included consent forms by language and practice type.

Language	Overall, n	Private practice, n	Nonprivate practice, n
Portuguese	25	21	4
English	21	15	6
Spanish	17	15	2
Italian	7	5	2
Turkish	4	4	0
French	3	3	0
Total	77	63	14

### Readability Assessment

The consent forms analyzed had an overall mean LIX score of 53 (SD 9), the mean number of words in each document was 3605 (SD 2226), the mean number of sentences was 283 (SD 255), and the mean number of words per sentence was 15 (SD 6). To illustrate the precision of the language-specific mean LIX estimates, 95% CIs were calculated and are presented in Table 3. As expected, CIs were wider for languages represented by fewer documents. The statistically significant differences observed in the 1-way ANOVA were confirmed by Welch ANOVA (*P*<.001), supporting the robustness of the overall findings despite the unequal sample sizes. There were no significant differences in the mean number of sentences (*P*=.35), mean number of words (*P*=.22), or mean number of words per sentence (*P*=.43). Differences between languages were statistically significant (1-way ANOVA, *P*<.001), confirmed by Welch ANOVA (*P*<.001). Exact values are presented in Table 3.

**Table 3.** Readability of online breast augmentation consent forms by language.

Language	Total, n	LIX^a^ score, mean (SD)	95% CI^b^	Sentence length (number of letters), mean (SD)	Total word count, mean (SD)	Words/sentence
Portuguese	25	55.3 (8.8)	51.7‐58.9	214 (180)	3074 (1952)	17.8
English	21	45.9 (6.7)	42.8‐49.0	380 (402)	3960 (1964)	14.5
Spanish	17	52.9 (7.9)	48.8‐56.9	275 (174)	3670 (2477)	17.7
Italian	7	57.6 (6.0)	52.0‐63.1	325 (110)	5358 (3176)	15.4
Turkish	4	62.5 (1.9)	59.5‐65.5	237 (76)	2716 (1059)	11.1
French	3	54.3 (3.5)	45.6‐63.1	188 (128)	3192 (2463)	15.5

a LIX: *Läsbarhetsindex*.

b CIs are shown only for the primary outcome (LIX score).

**Figure 1.**
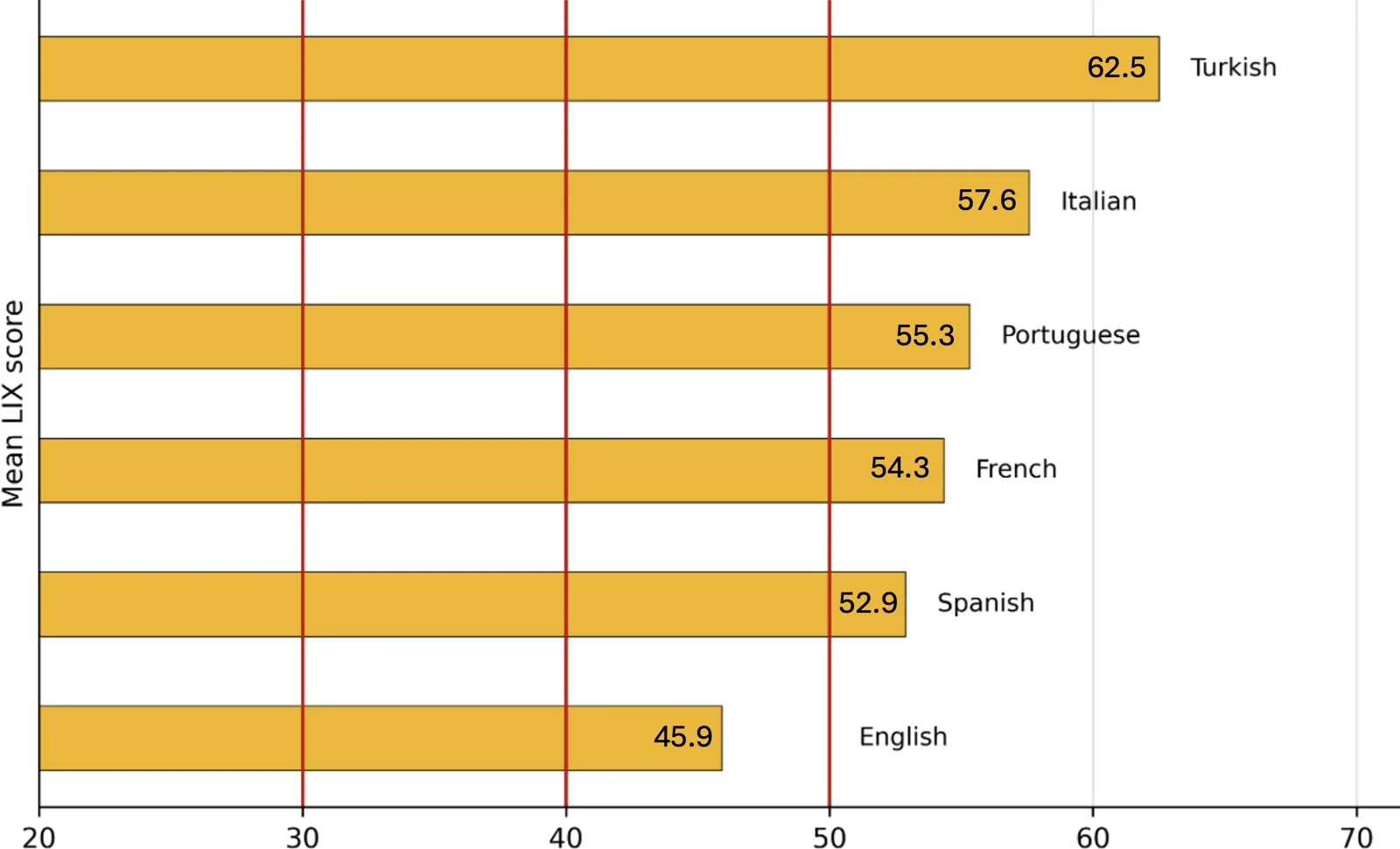
Readability of analyzed breast augmentation consent forms in the included languages. LIX: *Läsbarhetsindex*.

A text was classified as easy to comprehend if the mean LIX score was less than 30, as moderately difficult if the mean score was 30 to 39, as difficult if the mean score was 40 to 49, as very difficult if the mean score was 50 to 60, and as having very high linguistic complexity if the mean score was greater than 60. English (mean 46, SD 7), Spanish (mean 53, SD 8), and French (mean 54, SD 4) were the languages with the most readable breast augmentation consent forms. In contrast, Portuguese (mean 55, SD 9), Italian (mean 58, SD 6), and Turkish (mean 63, SD 2) were associated with the highest difficulty levels. None of the assessed texts had a LIX score below 40; therefore, no materials were categorized as easy or moderately difficult [19]. English fell into the difficult to understand category, while the remaining languages were classified as very difficult to understand [19]. Mean LIX values are shown in Figure 1.

### Readability, Consent Source, and Number of Consent Forms

There were no differences in the LIX scores (*P*=.19), number of sentences (*P*=.17), number of words (*P*=.27), or mean number of words in a sentence (*P*=.39) between consent forms issued by private practices and other providers. To investigate the relationship between the number of included consent forms in each language and the respective mean LIX scores, univariate linear regression was performed. There was no correlation between readability level and number of analyzed forms (*R^2^*=0.37, *P*=.2).

## Discussion

The analysis showed that the mean LIX score for preoperative informed consent forms exceeded 50 points. According to the classic interpretation of the LIX score proposed by Anderson [15] and used in contemporary analyses of medical materials [20], the following thresholds were assumed: less than 30 indicates an easy text, 30 to 39 a moderately difficult text, 40 to 49 a difficult text, 50 to 60 a very difficult text, and greater than 60 a text with very high linguistic complexity. An important consideration is that the analyzed documents were publicly available online consent forms rather than institution-specific forms collected directly from clinical practice. Consequently, the present findings should be interpreted as reflecting the readability of consent materials accessible to patients before consultation rather than the readability of documents routinely used during the formal consent process. Nevertheless, publicly available consent forms could influence patient expectations and understanding before meeting with a surgeon and therefore remain clinically relevant.

A score of 50 or greater obtained in this study suggests that the analyzed form should be classified as a very difficult text, significantly exceeding the level considered accessible to the average reader. In our previous study, it was demonstrated that values above 40 could already make consent too difficult to understand for people without specialized training [21].

Although the LIX index enables comparisons across different languages without requiring syllable counting, its interpretation should be considered in light of language-specific linguistic characteristics [15,16]. Moreover, LIX captures only 1 dimension of readability, primarily reflecting sentence length and the proportion of long words. It does not assess other important determinants of text comprehensibility, such as lexical familiarity, semantic complexity, discourse organization, or the use of domain-specific medical terminology. For example, Turkish is characterized by productive agglutinative morphology, whereby multiple grammatical morphemes are attached to a single lexical stem, naturally resulting in longer word forms than those typically observed in more analytic languages such as English [22]. Consequently, higher LIX scores could partly reflect typological differences in language structure rather than greater conceptual complexity for native speakers [22]. Therefore, the original LIX interpretation thresholds should be applied cautiously in multilingual studies, and future research should investigate language-specific calibration and validation of readability cut-off values.

The high linguistic complexity of informed consent documents could limit their actual comprehensibility, with significant ethical and legal consequences. Informed consent, as the foundation of patient autonomy, requires not only the formal provision of information but also the actual understanding of it. Overly complex language could make fully informed decisions about treatment very difficult.

The results of this study indicate a need to simplify the content of forms by shortening sentences, limiting specialized terminology, and adapting the language level to the standards recommended for the general population. Improving the readability of documents could enhance doctor-patient communication and more effectively implement the concept of informed consent.

In this study, English-language materials had lower LIX scores than those in other languages, indicating greater linguistic accessibility of the analyzed texts. The literature emphasized that the English-language digital space is one of the most developed areas of online health communication, and English speakers were among the most active consumers of medical information on the internet [23]. The large scale of internet use for health purposes promotes the development of audience-oriented communication standards. Although ANOVA demonstrated significant differences in mean LIX scores, language groups represented by only a few documents yielded less precise estimates, as reflected by wider CIs. Consequently, comparisons involving these languages should be interpreted with caution, and future studies including larger numbers of consent forms would improve the precision of language-specific estimates.

In addition, the development of social media as a channel for health communication introduced formats characterized by brevity and multimedia content. Microblogging platforms are structurally limited to short messages, which could influence how health information is presented [24]. A systematic review indicated that social media facilitates rapid information exchange and user interaction while also raising concerns about content quality and reliability [25]. Differences in readability between languages could therefore result from a combination of systemic, cultural, and communication factors rather than solely from the ownership model or market structure.

This analysis did not reveal any significant differences in readability between documents prepared by private entities and those prepared by implant manufacturers, scientific societies, or university hospitals. Regardless of their source, informed consent forms had similarly high LIX scores, indicating that they were difficult or very difficult to understand. This means that the problem of excessive linguistic complexity is systemic rather than sector-specific.

No correlation was found between the number of forms analyzed and their average readability level. This indicates that the problem was not the excessive diversity of the consent forms but rather their linguistic structure. In other words, increasing the number of consent forms does not improve the accessibility of the content. Instead, simplifying the document’s structure and language is key. This result suggests that it would be appropriate to develop a single, standardized, and linguistically simplified template for informed consent forms.

This study had several limitations. First, only publicly available online consent forms were analyzed. We did not verify whether these documents were identical to the consent forms routinely used in clinical practice, and therefore, they may not fully represent the documents signed by patients before surgery. Second, Google search results could vary over time and according to geographic location and search engine algorithms despite the use of private browsing mode [26]. In addition, the translated search terms were not formally validated by native speakers or professional translators, which could have influenced document retrieval in some languages. Third, readability was assessed using the LIX index, which evaluates linguistic complexity but does not directly measure patient comprehension or assess the completeness, accuracy, or clinical adequacy of informed consent content. Furthermore, although the LIX index has been successfully applied in multilingual readability research [15-17,19], its interpretation could be influenced by language-specific morphological and syntactic characteristics. Finally, the number of eligible consent forms differed substantially across languages, which could reduce the precision of language-specific estimates, particularly for languages represented by only a few documents. Only 1 eligible German-language form and 1 Hindi-language form were identified. Therefore, these languages were excluded from the comparative statistical analysis. This limited availability could reflect a genuine scarcity of publicly accessible documents or methodological factors, including search term selection, regional indexing, and local publication practices. Consequently, the findings should not be generalized to Germany or India, particularly given Germany’s high procedural volume for breast augmentation.

Although readability indices such as LIX provide an objective and reproducible measure of linguistic complexity, readability alone does not ensure that patients understand informed consent documents. Patient comprehension is also influenced by the patient’s health literacy, educational level, prior knowledge, cultural background, and anxiety and the quality of physician-patient communication [27-29]. Therefore, readability should be regarded as one component of informed consent quality rather than a direct measure of its effectiveness. Future studies should combine readability assessment with qualitative evaluation of content completeness and direct measures of patient comprehension, such as teach-back methods or validated comprehension questionnaires, to determine whether improved readability translates into better patient understanding and more informed decision-making [27-29].

To conclude, the publicly available online breast augmentation consent forms analyzed in this study exhibited high linguistic complexity, indicating that information resources accessible to prospective patients before consultation could be difficult to understand. Although these findings cannot be directly extrapolated to institution-specific consent forms used in routine clinical practice, they highlight the need to simplify the language and structure of online informed consent materials and to evaluate whether similar patterns exist in clinical consent documents.

## Abbreviations

ISAPS: International Society of Aesthetic Plastic Surgery
LIX: *Läsbarhetsindex*
